# Transcriptomic and Metabolomic Analyses Reveal That *Fusarium* sp. Isolate Is Associated with Growth Promotion in *Lolium perenne* L.

**DOI:** 10.3390/biology15191720

**Published:** 2026-09-30

**Authors:** Ganglian Liu, Jiahui Li, Yanwen Wang, Zhenghai Sun, Liping Li

**Affiliations:** 1 Yunnan International Joint R&D Center for Integrated Utilization of Ornamental Grass, College of Landscape and Horticulture, Southwest Forestry University, Kunming 650224, China; ganglianliu202608@163.com (G.L.); lijiahui990124@163.com (J.L.);; 2 College of Landscape and Horticulture, Southwest Forestry University, Kunming 650224, China; 3 College of Ecology and Environment (College of Wetlands), Southwest Forestry University, Kunming 650224, China

**Keywords:** *Fusarium* sp. isolate, growth-promoting effect, transcriptomics, metabolomics, *Lolium perenne* L.

## Abstract

Most previous studies have focused on the pathogenicity of *Fusarium* species towards crops. In this study, a *Fusarium* isolate was obtained from healthy *Chamelaucium uncinatum* plants; its ITS rDNA sequence showed 99% similarity to that of *Fusarium napiforme*. The fermentation preparation derived from this isolate promoted the growth and biomass accumulation of *Lolium perenne* L., a high-quality temperate forage grass; following seed treatment with the fermentation broth, the germination rate increased from 61.5% to 82%. In the pot experiment, in which the fermentation preparation was applied by root irrigation, plant height and root length of the host plants increased significantly by 20.0% and 30.6% (*p* < 0.05) and dry weight increased by 67.6% (*p* = 0.010), whereas fresh weight showed a non-significant increasing trend (+22.5%, *p* = 0.136); no disease symptoms were observed. Integrated transcriptomic and metabolomic analyses showed that exposure to the fermentation broth was associated with changes in genes and metabolites related to biomass accumulation. This study identified a candidate fungal isolate for the future development of microbial inoculants for forage crops.

## Simple Summary

This study aims to address a pressing global issue: the excessive use of chemical pesticides and fertilisers has led to widespread soil contamination and the destruction of natural ecosystems; pathogenic fungi have long been regarded solely as harmful to crop growth, and their potential as beneficial microorganisms within healthy plants remains largely untapped. We screened healthy Chamelaucium uncinatum plants for a growth-promoting fungus that did not cause visible disease symptoms, to test its effect on *Lolium perenne* L.—a widely used forage crop—and to reveal how this fungus promotes better plant growth without causing harm. A fungal fermentation broth prepared from this strain increased the germination rate of *Lolium perenne* L. seeds from 61.5% to 82% and increased the final dry weight of the grass by 67.6%, whilst no disease symptoms were observed. In-depth transcriptomic and metabolomic analyses revealed that the fungal fermentation broth altered the plants’ transcriptional and metabolic profiles. This strain may be valuable for the future development of microbial inoculants or potential biofertilisers.

## 1. Introduction

Since the 20th century, the widespread use of chemical pesticides and fertilisers has led to severe soil pollution, resulting in the degradation of ecosystems worldwide. In order to effectively reduce environmental pollution and even restore the environment, biofertilisers and biological agents for agricultural use have gradually been developed [1]. Among these, endophytic fungi found within plants are considered promising sources for the development of such products. Endophytic fungi are a class of fungi capable of surviving long-term in specific parts of plants without affecting their growth and development; they maintain a close symbiotic relationship with their host plants [2,3]. Several well-characterised endophytic fungi confer specific benefits on their hosts: Serendipita indica promotes nutrient uptake and growth in a range of hosts [4]; *Epichloë festucae var. lolii* enhances stress tolerance in *Lolium perenne* [5]. Currently, pathogenic fungi are typically studied for their adverse effects on crop yield and quality. The presence of such fungi disrupts the growth and development of the host plant [6,7]. It has been shown that endophytic entomopathogenic fungi can promote growth and enhance resistance to adverse environmental conditions [8]. Until recently, the beneficial effects of endophytic fungi on host growth and development received considerably less attention than their pathogenic effects.

As important members of the plant microbiome, endophytic fungi are abundant in various plants. Most of them originate from microbe-rich soil, air, and water, or are attached to the plant’s internal microecosystem [9]. Through natural transmission routes, external microorganisms can be carried into plants by wind, water, or other organisms, where they may colonise specific tissues and establish stable associations with the host [10]. The fact that *Alternaria* sp. parasitises *Borrichia frutescens* (L.) via the biological vector Asphondylia borrichiae Rossi & Strong further corroborates this [11]. The capacity of different parts of a plant to harbour endophytic fungi also varies to some extent. Generally speaking, the diversity of endophytic fungi in a plant’s root system is far greater than that in its above-ground parts. Furthermore, endophytic fungal communities in the above-ground parts of plants vary in response to changes in light intensity and temperature fluctuations [12,13]. Research has shown that certain endophytic bacteria, when present in the seed coats of *Abelmoschus esculentus*, possess properties that both promote plant growth and alleviate salt stress [14]. In natural environments, since most of the phosphorus in the soil exists in the form of insoluble inorganic phosphates and other organic phosphorus compounds, plants are unable to absorb it normally, leading to phosphorus deficiency symptoms. However, endophytic fungi secrete a variety of organic acids and enzymes to break down these insoluble substances, thereby making it easier for plants to absorb phosphorus from the soil. Strains of Bacillus stercoris and Enterobacter quasihormaechei have been found to secrete auxins to promote plant growth whilst solubilising phosphorus [15]. In well-documented mutualistic associations, for example that between Piloderma croceum and *Quercus robur* L., the fungus enhances nutrient uptake, mobilises insoluble nutrients and promotes plant growth [16]. *Burkholderia gladioli DJB4-8* promotes plant growth by converting insoluble phosphorus in the soil whilst also altering the photosynthetic physiology and endogenous IAA levels in maize plants [17]. Symbiotic fungi can also enhance the host plant’s ability to absorb nutrients; in *Brassicaca rapa var. parachinensis*, the symbiotic *Tinctoporellus* species isolate *AR8* led to an increase in plant biomass by boosting the production of secondary metabolites in the plant’s phenylpropanoid metabolic pathway [18]. These secondary metabolites are structurally similar to hormones produced by the plants themselves and can influence the host plant’s signal transduction pathways [19,20,21]. These factors can affect the host plant’s cell division, organ formation, and developmental processes, thereby promoting plant growth and enhancing its growth potential [22]. Research has shown that *Fusarium avenaceum* can produce gibberellin A2 and zeatin; when herbaceous plants such as ryegrass are inoculated with these endophytic fungi, there is a significant increase in their root volume, surface area and number of root tips [23]. These microorganisms are also capable of synthesising plant hormones such as gibberellins (GA) and cytokinins (CTK), thereby regulating the growth and development of both the above-ground and below-ground parts of the plant [24,25].

*Lolium perenne* L. (*L. perenne*) is widely used in temperate agricultural systems around the world and is of significant value to global agriculture [26]. It serves as a primary source of high-quality forage during winter and spring, effectively bridging the forage gap in temperate regions and providing the global livestock industry with high-quality, highly digestible forage, thereby significantly reducing livestock production costs [27,28]. At the same time, it is an important ecological cover crop: its well-developed fibrous root system effectively intercepts surface runoff and prevents soil erosion [29]. In the process of reclaiming saline-alkali land, planting *L. perenne* can reduce the salinity of the topsoil and rapidly increase soil organic matter content after tillage [30]. *L. perenne* is a plant with unique biological characteristics and ecological adaptations. Owing to its ability to interact with fungi, it is frequently used in studies investigating endophytic fungi that promote growth and enhance stress tolerance [31]. In 2000, Malinowski and Belesky, in their study on the mechanisms by which endophytic fungi influence mineral nutrient uptake in herbaceous plants, noted that the root system of *L. perenne* undergoes significant changes when infected by endophytic fungi [32]. Furthermore, under the regulation of *Trichoderma asperellum* 152-42, changes occur in the plant hormone and carbon–nitrogen metabolism pathways in *L. perenne*, thereby enhancing its resistance to adverse environmental conditions [33]. In 2015, Dupont employed transcriptomic methods to investigate *L. perenne* under endophytic fungal symbiosis, revealing that the plant exhibited highly differential gene expression in secondary metabolite synthesis, changes in antioxidant enzyme activity, and ion transport [34].

A further consideration for the application of any microorganism in plant cultivation is its ability to become established in soil that already harbours a naturally balanced and highly competitive microbial community. Introduced strains must compete with the resident microflora for nutrients and space, and may be subject to antagonistic interactions, including the production of antimicrobial compounds, competition for iron and other limiting resources, and mycoparasitism. Previous studies have found that the antagonistic relationship between fungi and bacteria gradually evolves into a symbiotic relationship over time [35]. Under drought stress, the interaction between *arbuscular mycorrhizae* and biochar enhances pasture soil fertility [36]. In field peas, however, *arbuscular mycorrhizal* fungi, once established on the roots, lead to a reduction in the germination rate of seeds belonging to the genera *Orobanche* and *Phelipanche* [37]. Consequently, adaptation to the recipient soil environment, rather than intrinsic plant growth-promoting capacity alone, is now recognised as one of the principal bottlenecks in the development of microbial inoculants. These aspects therefore represent an important direction for future work, in which colonisation dynamics, interactions with the native soil microflora and performance under field conditions should be examined.

In recent years, research into the role of endophytic fungi in promoting plant growth has primarily focused on overall plant traits and traditional physiological and biochemical aspects. Most researchers have assessed these effects mainly by measuring plant height, root development or changes in biomass, whilst utilising chlorophyll content and antioxidant enzyme activity in leaves to elucidate the underlying mechanisms of action [38,39,40]. Currently, plant fertilisers are evolving towards more environmentally friendly, bio-based alternatives; consequently, this study focuses on the screening of high-quality endophytic fungi isolated from *Chamelaucium uncinatum* (*C. uncinatum*). The selected fungus may have potential for promoting plant growth, offering a candidate resource for research into microbial inoculants. This study provides a systematic investigation of the fungi associated with *C. uncinatum* and has enriched the repository of unique microbial strains in the field of plant microbiology. In this study, *L. perenne* was exposed to selected high-quality endophytic fungal fermentation broths, and phenotypic changes in *L. perenne* were observed. Using *L. perenne* as the primary subject of analysis, high-throughput transcriptomics and metabolomics methods were employed to investigate the effects of these high-quality *C. uncinatum* endophytes on *L. perenne* from the phenotypic to the molecular level. This study has not only deepened our understanding of how endophytic fungi promote plant growth, but has also provided candidate molecular pathways for how microorganisms may promote the growth of different hosts.

## 2. Materials and Methods

### 2.1. Experimental Materials

*C. uncinatum* explants used in this study were sourced from the greenhouse at the Arboretum of Southwest Forestry University. The plants used were fully mature and free from disease and pests. The fungal isolate obtained from these C. uncinatum explants was preserved at the Landscape and Horticulture Laboratory of Southwest Forestry University. The *L. perenne* seeds used in this study were stored at 4 °C in the Landscape and Horticulture Laboratory of Southwest Forestry University.

### 2.2. Molecular Identification of the Fungal Isolate

In this study, the ITS rDNA method was employed for the molecular identification of the isolated fungus. The Magen Fungal DNA Extraction Kit (D3171-02, Guangzhou Magen Biotechnology Co., Ltd., Guangzhou, China) was used to extract fungal DNA. PCR amplification was performed using the universal fungal primers ITS1 and ITS4, and the undiluted PCR products were sent to Qingke Biotechnology Co., Ltd. (Kunming, China) for sequencing. The obtained sequences were uploaded to the NCBI website (https://www.ncbi.nlm.nih.gov, URL accessed on 25 April 2025) for homology analysis, enabling a preliminary determination of the taxonomic status of the isolate.

### 2.3. Preparation of the Fermentation Preparation

In this study, the test strains were inoculated into PDB liquid medium and incubated at 28 °C, at 180 r∙min^−1^ on a constant-temperature shaking incubator for 4 days. The fermentation broth was resuspended by shaking and diluted with sterile water; a haemocytometer was used to adjust the spore concentration to approximately 1 × 10^6^ CFU/mL, thereby yielding the fermentation preparation used in all subsequent experiments.

### 2.4. Experiments on the Promotion of Plant Germination by Fungal Fermentation Preparation

Plump, uniformly sized seeds were selected for the experiments. The sterilised seeds were placed in a fermentation suspension of the fungal strain and shaken at 180 r∙min^−1^ and 28 °C for 24 h. Under identical conditions, an equal volume of sterile PDB liquid medium, containing no fungal cells or fungal metabolites, was used as the germination control (designated CK-G; a medium-only control). The fermentation treatment is designated E1. After soaking, the seeds of both groups were transferred to sterile Petri dishes lined with two layers of sterile filter paper moistened with sterile water. Each treatment group comprised four replicates, with 50 seeds per replicate. Seeds that had been incubated for 24 h were used for the germination experiment. The seeds were sown onto sterile Petri dishes lined with two layers of sterile filter paper; the filter paper was moistened with sterile water, and the dishes were incubated in a constant-temperature incubator at 28 °C.

### 2.5. Experiment on the Promotion of Plant Growth by Fungal Fermentation Broth

In this experiment, the germinated seedlings from Section 2.4 were transplanted into sterilised plastic pots (top diameter 12 cm, bottom diameter 10 cm, height 15 cm), each pot containing 1.2 kg of autoclaved seedling growing medium (peat: perlite: vermiculite = 3:1:1 (by volume), pH 6.45, organic matter 248.6 g/kg, total nitrogen 12.3 g/kg, available phosphorus 35.7 mg/kg, available potassium 168.2 mg/kg). One germinated seedling was transplanted into each pot; each pot therefore contained a single plant and represented one biological replicate, with 10 replicates per treatment group. Plants were cultivated in a controlled growth chamber at 25/20 °C (day/night), with a 16 h photoperiod (photosynthetic photon flux density 350 μmol·m^−2^·s^−1^) and relative humidity of 60–70%. Pots were arranged according to a completely randomised design and rearranged weekly. Plants were watered with 100 mL of tap water every two days to maintain soil moisture content at approximately 60% of field capacity; a half-strength Hoagland solution was applied every two weeks at a rate of 50 mL per pot. From the two-leaf stage onwards, a fungal fermentation suspension (1 × 10^6^ CFU/mL, 150 mL per pot per application) was applied via root irrigation every seven days. The pot control (designated CK) was treated with an equal volume of sterile water at the same time points. To avoid confusion with the germination control (CK-G), the pot control is referred to as CK-P throughout; the label “CK” denotes this pot control (CK). On the third day after the fourth irrigation (day 30 of the growth cycle), 10 plants were sampled from each group to measure morphological parameters. Plant height and root length were measured for all 10 biological replicates in each treatment group; fresh weight and dry weight were measured using 5 biological replicates from each treatment group. The remaining five plants per treatment were reserved for the transcriptomic and metabolomic analyses described in Section 2.6 (three used, two stored); the plants used for destructive biomass measurement and those used for molecular analysis were therefore different individuals.

### 2.6. Methods for the Preparation of Samples Used in Transcriptomic and Metabolomic Analyses

In this study, root and leaf tissues were collected separately. For the transcriptomic and metabolomic analyses, five plants per treatment were reserved (Section 2.5). Three of these plants per treatment were used for the transcriptome and metabolome analyses (*n* = 3), and the remaining two plants per treatment were flash-frozen at −80 °C and retained as spares for future use. Each of the three analysed samples was a whole individual plant grown in a single pot; no tissue from different plants was pooled, so the three biological replicates were independent. The experiment comprised a control group (CK) and a treatment group (E1), with root samples labelled GCK and GE1, and leaf samples labelled YCK and YE1; a fully matched analytical strategy was adopted for the transcriptome and metabolome analyses, with both sets of assays conducted simultaneously on the same biological replicate samples. Each tissue sample was divided into two sub-samples, one for RNA sequencing and the other for metabolite extraction, ensuring a one-to-one correspondence between the two sets of data; all samples were collected on the 30th day of plant growth, i.e., three days after the fourth root irrigation treatment. Immediately after collection, the samples were rapidly frozen in liquid nitrogen and subsequently stored in an ultra-low temperature freezer at −80 °C until subsequent nucleic acid and metabolite extraction procedures.

### 2.7. Transcriptome Sequencing

RNA was extracted from the samples using the Omega Plant RNA Extraction Kit (Omega Bio-Tek, Shanghai, China) according to the manufacturer’s instructions. EachRNA sample was assayed for RNA concentration using a nucleic acid protein instrument and frozen at −80 °C. mRNA was enriched with poly A structure in total RNA using oligo(dT) magnetic beads, and ionic disruption was used to break the RNA into fragments about 300 bp in length. First-strand cDNA was synthesized using a 6-base random primer and reverse transcriptase using RNA as a template for a 300 bp fragment. The first-strand cDNA was used as a template for the synthesis of the second-strand cDNA. Once the library construction was complete, PCR amplification was used to enrich the library for fragments, followed by library selection based on fragment size, which was 450 bp. Subsequently, the libraries underwent quality assessment via an Agilent 2100 Bioanalyzer (Agilent Technologies, Santa Clara, CA, USA), after which the total concentration and effective concentration of the libraries were evaluated. Subsequently, the libraries comprising disparate index sequences were combined in a proportionate manner, contingent on the effective concentration of the libraries and the quantity of data required from them. The mixed libraries were uniformly diluted to 2 nM, and single-stranded libraries were formed by base denaturation. Following the extraction, purification, and construction of the libraries, the libraries were subjected to paired-end (PE) sequencing using next-generation sequencing (NGS) based on the Illumina NovaSeq 6000 (Illumina, Shanghai, China) sequencing platform. Differentially expressed genes (DEGs) were identified using the criteria of |log_2_FC| ≥ 1 and FDR < 0.01 [41,42,43,44,45]. For specific transcriptomic and analytical parameters, see Table A1 and Table A2.

### 2.8. Metabolomic Sequencing

#### 2.8.1. Sample Preparation and Extraction

Biological samples were freeze-dried in a vacuum freeze-dryer (Nanjing Feilong Pharmaceutical Equipment Co., Ltd., Kunming, China). The freeze-dried sample (50 mg) was dissolved in 1.0 mL of extraction solvent (methanol:acetonitrile:water = 1:2:1) and vortexed for 30 s. The mixture was homogenised in a mixer mill with a bead for 10 min at 45 Hz, and sonicated in an ice-water bath for 10 min. The samples were then kept at −20 °C for 1 h. After centrifugation at 4 °C and 12,000× *g* rpm for 15 min, 300 µL of the supernatant was filtered (0.22 µm) into a 2 mL injection vial, and 10 µL of each sample was pooled to prepare quality-control (QC) samples before UPLC-MS/MS analysis.

#### 2.8.2. UPLC Conditions

The sample extracts were analyzed using a UPLC-ESI-MS/MS system (UPLC, Waters Acquity I-Class PLUS; MS, Applied Biosystems QTRAP 6500+). The analytical conditions were as follows: UPLC column; Waters HSS-T3 (1.8 µm, 2.1 × 100 mm). The mobile phase consisted of solvent A, pure water with 0.1% formic acid and 5 mM ammonium acetate, and solvent B, acetonitrile with 0.1% formic acid. Sample measurements were performed with a gradient program that employed the starting conditions of 98% A, 2% B and kept for 1.5 min. Within 5.0 min, a linear gradient to 50% A, 50% B was programmed; within 9.0 min, a linear gradient to 2% A, 98% B was programmed, and a composition of 2% A, 98% B was kept for 1 min. Subsequently, a composition of 98% A, 2% B was adjusted within 1 min and kept for 3 min. The flow rate was set at 0.35 mL∙min^−1^; the column oven was set to 50 °C; and the injection volume was 4 µL. The effluent was alternatively connected to an ESI-triple quadrupole-linear ion trap (QTRAP)-MS.

#### 2.8.3. ESI-QTRAP-MS/MS

The ESI source operation parameters were as follows: source temperature 550 °C; ion spray voltage (IS) 5500 V (positive ion mode)/−4500 V (negative ion mode); ion source gas I (GSI), gas II (GSII), curtain gas (CUR) were set at 50, 55, and 35 psi, respectively; the collision-activated dissociation (CAD) was medium. Instrument tuning and mass calibration were performed with 10 and 100 μmol/L polypropylene glycol solutions in QQQ and LIT modes, respectively. QQQ scans were acquired as MRM experiments with collision gas (nitrogen) set to medium. DP (declustering potential) and CE (collision energy) were further optimised for individual MRM transitions. A specific set of MRM transitions was monitored for each period according to the metabolites eluted within this period.

#### 2.8.4. PCA and Spearman Correlation Analysis

After normalizing the original peak area information with the total peak area, the follow-up analysis was performed. Principal component analysis and Spearman correlation analysis were used to judge the repeatability of the samples within the group and the quality control samples.

#### 2.8.5. Selection of Differentially Accumulated Metabolites

According to the grouping information, fold changes were calculated and compared, and a *t*-test was used to calculate the *p* value for each compound. The R package ropls was used to perform OPLS-DA modeling, and 200 permutation tests were performed to verify the reliability of the model. The VIP value of the model was calculated using multiple cross-validation. The method of combining the difference multiple, the *p* value and the VIP value of the OPLS-DA model was adopted to screen the differential metabolites.

#### 2.8.6. Database Annotation and KEGG Enrichment Analysis

The identified compounds were searched for classification and pathway information in KEGG (http://www.kegg.jp/kegg/compound/, URL accessed on 27 April 2025), HMDB (https://hmdb.ca/metabolites/, URL accessed on 27 April 2025) and lipidmaps (https://lipidmaps.org/, URL accessed on 27 April 2025) databases. The difference metabolites of KEGG pathway (http://www.kegg.jp/kegg/pathway.html, URL accessed on 27 April 2025) enrichment significance were calculated using hypergeometric distribution test.

#### 2.8.7. Metabolomic Analysis Parameters

For specific metabolomics parameters and analytical parameters, see Table A3.

### 2.9. Statistical Analysis

#### 2.9.1. Germination Experiment

The Petri dish was treated as the experimental unit, with 50 seeds per dish serving as subsamples. To properly account for the hierarchical data structure and avoid pseudoreplication, germination data were analysed using a generalised linear mixed model (GLMM) with a binomial error distribution and logit link function. Treatment (E1 vs. CK-G) was included as a fixed effect, and Petri dish identity was included as a random intercept. The model was fit using the lme4 package in R v4.3.1. Statistical significance of the treatment effect was assessed via likelihood ratio tests comparing the full model to a reduced model without the treatment term. Effect sizes are reported as odds ratios with 95% profile likelihood confidence intervals.

#### 2.9.2. Pot Experiment (Phenotypic Traits)

Differences between the fungal treatment (E1) and control (CK) were tested using Welch’s two-sample *t*-test, which does not assume equal variances. Normality was assessed via Shapiro–Wilk tests and Q–Q plots; homogeneity of variances was evaluated with Levene’s test. Effect sizes are reported as Cohen’s d with 95% confidence intervals, calculated using the pooled standard deviation. All statistical analyses were performed in R v4.3.1 using the rstatix and effsize packages. Exact *p*-values are reported throughout; *p* < 0.05 was considered statistically significant.

## 3. Results

### 3.1. Molecular Biological Characterization

**Figure 1:**
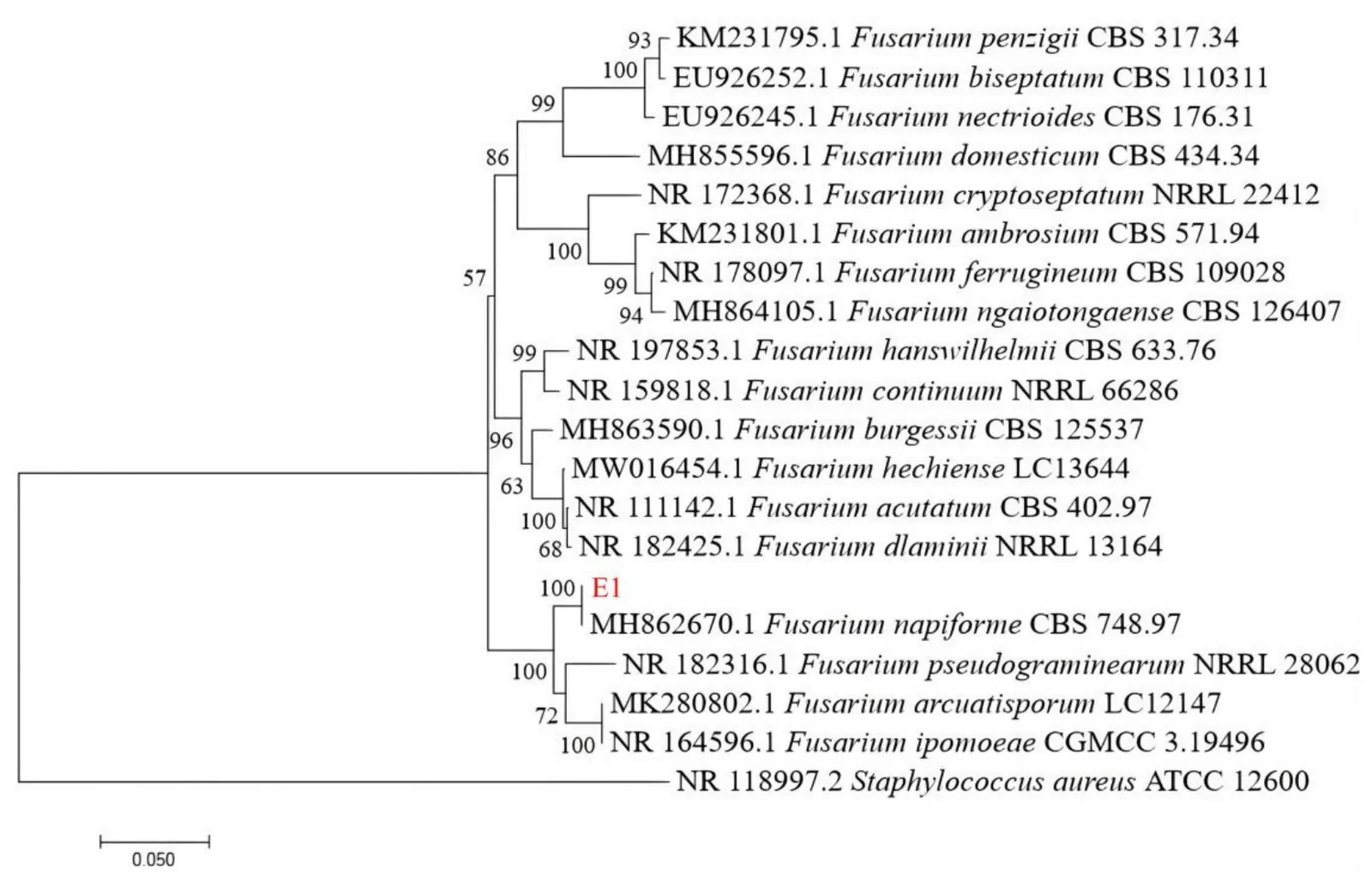
Phylogenetic tree of evolution.

**Figure 2:**
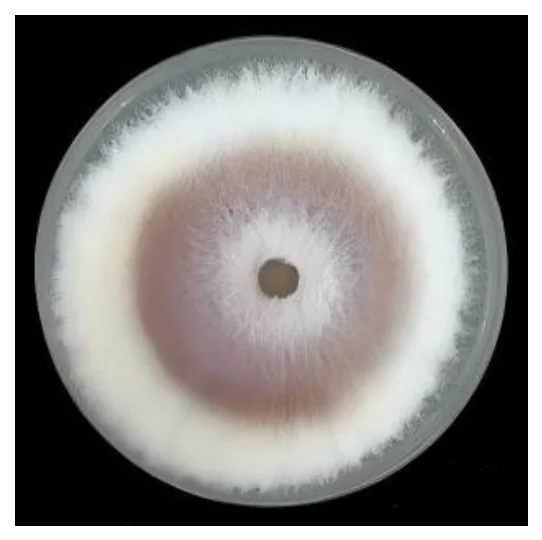
Spore-forming structures.

This study constructed a phylogenetic tree for the fungal isolate obtained from *C. uncinatum*. The obtained sequences were uploaded to the NCBI website (https://www.ncbi.nlm.nih.gov, URL accessed on 25 April 2025) for homology alignment to preliminarily determine the taxonomic status of each sample. A phylogenetic tree was constructed using MEGA 7.0 to confirm the taxonomic status of this fungus. As shown in Figure 1, isolate E1 clustered with Fusarium napiforme with 99% ITS rDNA sequence similarity. The spore-bearing structures of this fungus are shown in Figure 2. ITS rDNA alone does not resolve species within the *Fusarium fujikuroi* species complex; the isolate is therefore referred to as *Fusarium* sp. isolate pending multi-locus sequencing (TEF1-α, RPB2).

### 3.2. Measurement of Physiological Parameters

In this study, the seed germination rate in the control group was 61.5%; whereas, following treatment with a suspension of *Fusarium* sp. isolate fermentation, the seed germination rate in the experimental group reached 82%, which was significantly higher than that of the control group (Table A4). This study compared growth parameters between the fungal treatment group and the blank CK control group. Ten biological replicates were set up for each group; plant height and root length were analysed using statistics for the entire sample set, whilst fresh weight and dry weight were measured on five biological replicates per treatment (*n* = 5). The results showed that the plants in the fungal treatment group had an average plant height of 33.80 cm, an average root length of 10.08 cm, an average fresh weight of 0.90 g and an average dry weight of 0.09 g, whilst plants in the CK control group had an average plant height of 28.17 cm, an average root length of 7.72 cm, an average fresh weight of 0.74 g and an average dry weight of 0.05 g. Compared with the CK control group, the fungal treatment group showed increases of 20.0%, 30.6%, 22.5% and 67.6% in plant height, root length, fresh weight and dry weight, respectively (Table A5). This indicates that this fungal fermentation broth significantly increased plant height, root length and dry weight, whilst fresh weight showed a non-significant increasing trend (*p* = 0.136) of the test plants, providing direct phenotypic data support for the subsequent development of growth-promoting microbial preparations.

### 3.3. Transcriptome Analysis

#### 3.3.1. Differential GO Enrichment Analysis of the Transcriptome

**Figure 3:**
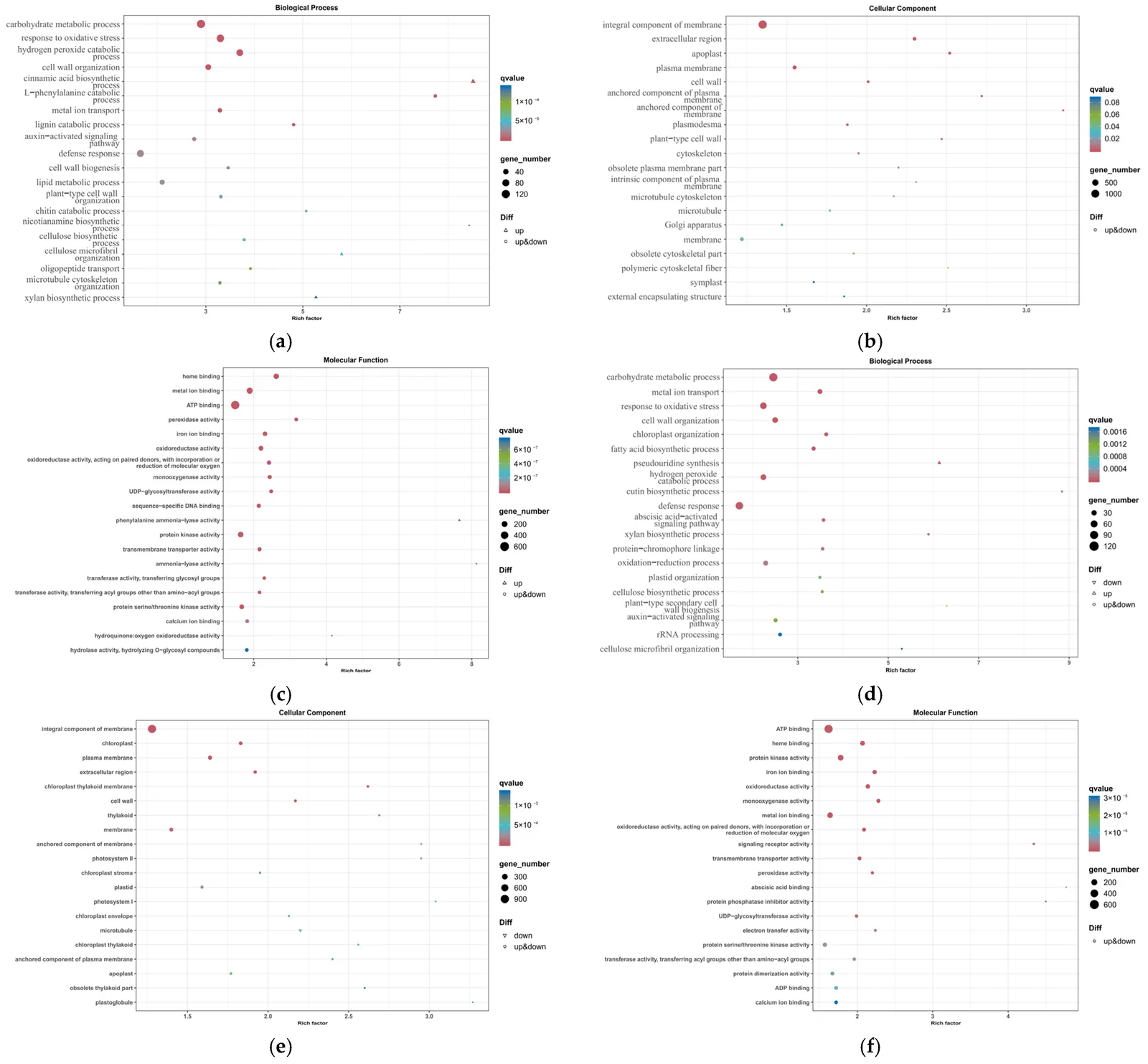
Differential Expression GO Enrichment Analysis. Note: GO enrichment analysis of the root system: (**a**) Biological Process. (**b**) Cellular Component. (**c**) Molecular Function. GO enrichment analysis of the leaf system: (**d**) Biological Process. (**e**) Cellular Component. (**f**) Molecular Function. CK, control; E1, fermentation-broth treatment; G, root tissue; Y, leaf tissue; *n* = 3 independent biological replicates per treatment (each an individual plant). DEGs were defined as |log_2_FC| ≥ 1 and FDR < 0.01.

GO functional enrichment analysis was performed on plant root tissue samples, and several DEGs potentially involved in plant growth and development were identified by comparing the transcriptomic data against the reference genome. Within the ‘Biological Process’ functional category, the functional categories with the highest levels of gene differentiation in the roots were as follows: ‘carbohydrate metabolic process’, ‘response to oxidative stress’, ‘hydrogen peroxide catabolic process’, ‘cell wall organisation’ and ‘cinnamic acid biosynthetic process’ (Figure 3a). Among these, the ‘carbohydrate metabolic process’ category contained the highest number of DEGs, totalling 158; this was followed by the ‘response to oxidative stress’ category with 122 DEGs; the ‘hydrogen peroxide catabolic process’ category contained 98 DEGs; 71 DEGs were identified in the ‘cell wall organisation’ category; and 21 up-regulated DEGs were identified in the ‘cinnamic acid biosynthetic process’ category. Within the ‘Cellular Component’ functional category, the functional categories with the highest levels of gene differential expression in the root system were as follows: ‘integral component of membrane’, ‘extracellular region’, ‘apoplast’, ‘plasma membrane’ and ‘cell wall’. Among these, ‘integral component of membrane’ showed the highest enrichment of DEGs, with a total of 797 differentially up-regulated genes; this was followed by the ‘extracellular region’ functional category, with 69 differentially up-regulated genes; the ‘apoplast’ category contained 2 DEGs; one differentially up-regulated gene was identified in the ‘plasma membrane’ category; and 32 differentially up-regulated genes were identified in the ‘cell wall’ category (Figure 3b). Within the ‘Molecular Function’ category, there was a high degree of enrichment of DEGs in the following functions: haem binding, metal ion binding, ATP binding, peroxidase activity and iron ion binding (Figure 3c). There were 253 DEGs in the ‘haem binding’ functional category; 329 were identified in the ‘metal ion binding’ category; 676 were found in the ‘ATP binding’ category; 103 were identified in the ‘peroxidase activity’ category; and 163 were identified in the ‘iron ion binding’ category.

In plant leaves, GO enrichment analysis of the transcriptome revealed that, within the ‘Biological Process’ functional category, DEGs were highly enriched in the following processes: carbohydrate metabolic process, metal ion transport, response to oxidative stress, cell wall organization, chloroplast organization (Figure 3d). In the ‘carbohydrate metabolic process’ function, 120 DEGs were enriched; in the ‘metal ion transport’ function, there were 37 DEGs; in the ‘response to oxidative stress’ function, there were 74 DEGs; the ‘cell wall organisation’ and ‘fatty acid biosynthetic process’ functions showed slightly lower enrichment levels, with 52 and 27 DEGs respectively. Within the ‘Cellular Component’ functional category, the ‘integral component of membrane’ showed the highest enrichment of DEGs, with a total of 1159 genes; this was followed by ‘chloroplast’, with 146 genes; the ‘extracellular region’ showed an enrichment of 118 DEGs; the ‘plasma membrane’ contained 190 DEGs; and the ‘chloroplast thylakoid membrane’ showed a lower level of enrichment, with a total of 49 DEGs (Figure 3e). Within the ‘Molecular Function’ category, the functions showing the highest levels of enrichment are ATP binding, haem binding, protein kinase activity, iron ion binding and oxidoreductase activity (Figure 3f).

#### 3.3.2. Differential KEGG Enrichment Analysis of the Transcriptome

Based on the functional annotation results of DEGs, KEGG pathway enrichment analysis was employed to investigate the effects of the fermentation preparation on *L. perenne* and its mechanism of action. The results indicated that, following root irrigation with the fermentation preparation, a total of 2406 DEGs in root tissue were involved in 134 pathways, whilst 2092 DEGs in leaf tissue were involved in 134 pathways. The number of annotated genes in root tissue was significantly higher than that in leaf tissue, with a difference of approximately 314 DEGs.

**Figure 4:**
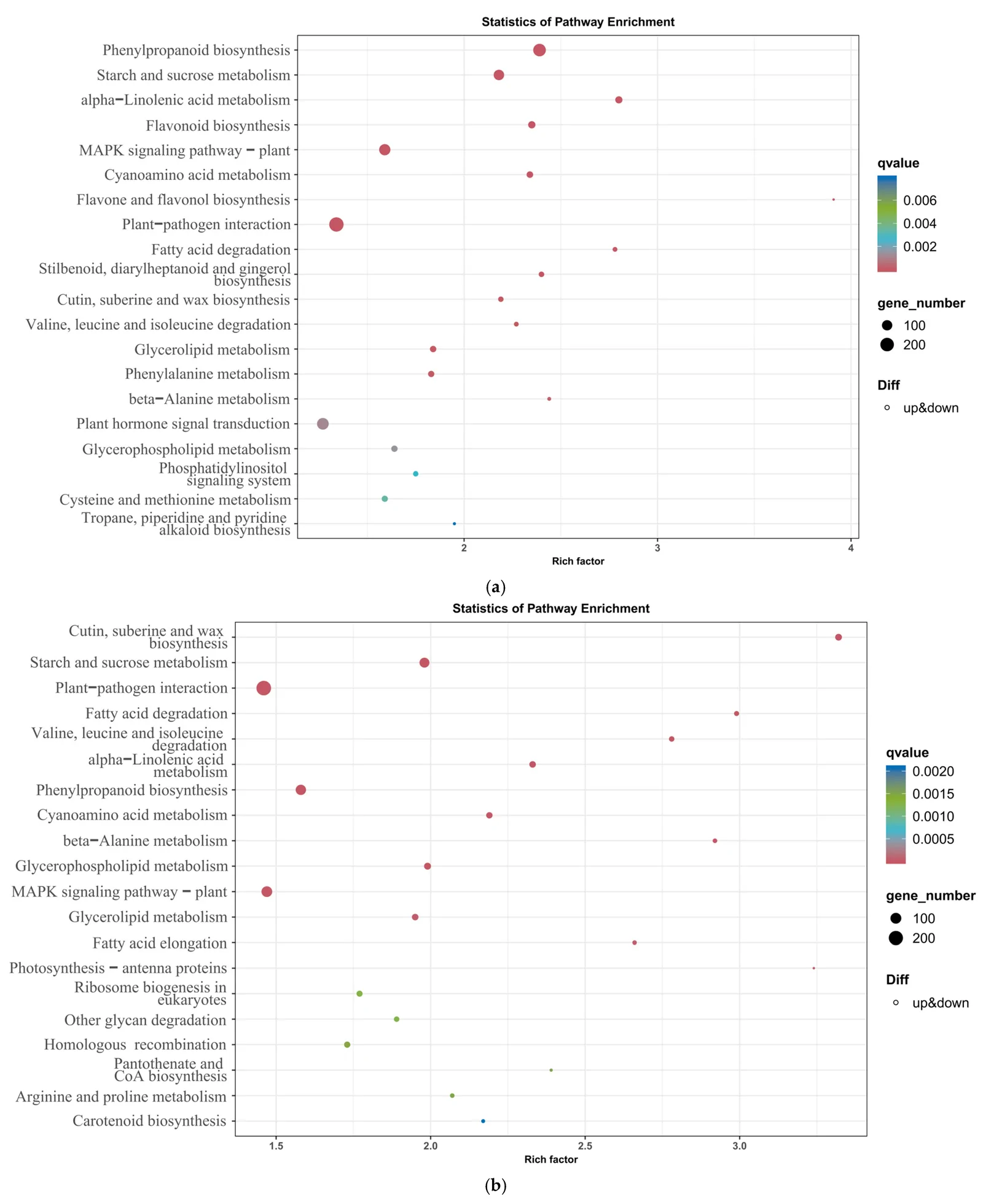
Differential Expression of Genes Enriched in KEGG. (**a**) KEGG Enrichment Analysis of Root-Specific Genes. (**b**) KEGG Enrichment Analysis of DEGs in Leaves. CK, control; E1, fermentation-broth treatment; G, root tissue; Y, leaf tissue; *n* = 3 independent biological replicates per treatment (each an individual plant). DEGs were defined as |log_2_FC| ≥ 1 and FDR < 0.01.

Enrichment analysis of DEGs in plant root metabolic pathways revealed that genes in pathways such as phenylpropanoid biosynthesis, starch and sucrose metabolism, α-linolenic acid metabolism, flavonoid biosynthesis, and the MAPK signalling pathway–plant were highly significant (Figure 4a). A total of 225 DEGs were enriched in the phenylpropanoid biosynthesis pathway, comprising 107 up-regulated genes and 118 down-regulated genes; furthermore, the ‘Starch and sucrose metabolism’ pathway contained a total of 75 downregulated genes and 73 upregulated genes; 65 DEGs were enriched in the ‘alpha-linolenic acid metabolism’ pathway. The ‘Flavonoid biosynthesis’ and ‘MAPK signalling pathway—plant’ pathways contained 67 and 174 DEGs, respectively. Analysis of the plant root transcriptome revealed that a large number of genes that were significantly up- or down-regulated were predominantly associated with functions such as plant hormone signalling, carbohydrate metabolism and the biosynthesis of secondary metabolites.

The pathway showing the most significant enrichment of DEGs in the leaves was ‘Cutin, suberin and wax biosynthesis’, in which a total of 50 DEGs were enriched. This was followed by ‘Starch and sucrose metabolism’, ‘Plant–pathogen interaction’, ‘Fatty acid degradation’ and ‘Valine, leucine and isoleucine degradation’ (Figure 4b). Among these, 117 DEGs were enriched in the ‘Starch and sucrose metabolism’ pathway; 282 DEGs were annotated in the ‘Plant–pathogen interaction’ pathway; 29 genes were annotated in the ‘Fatty acid degradation’ pathway; and 33 DEGs were found in the ‘Valine, leucine and isoleucine degradation’ pathway.

### 3.4. Metabolomic Analysis

#### 3.4.1. Metabolomics Data Quality Control

**Figure 5:**
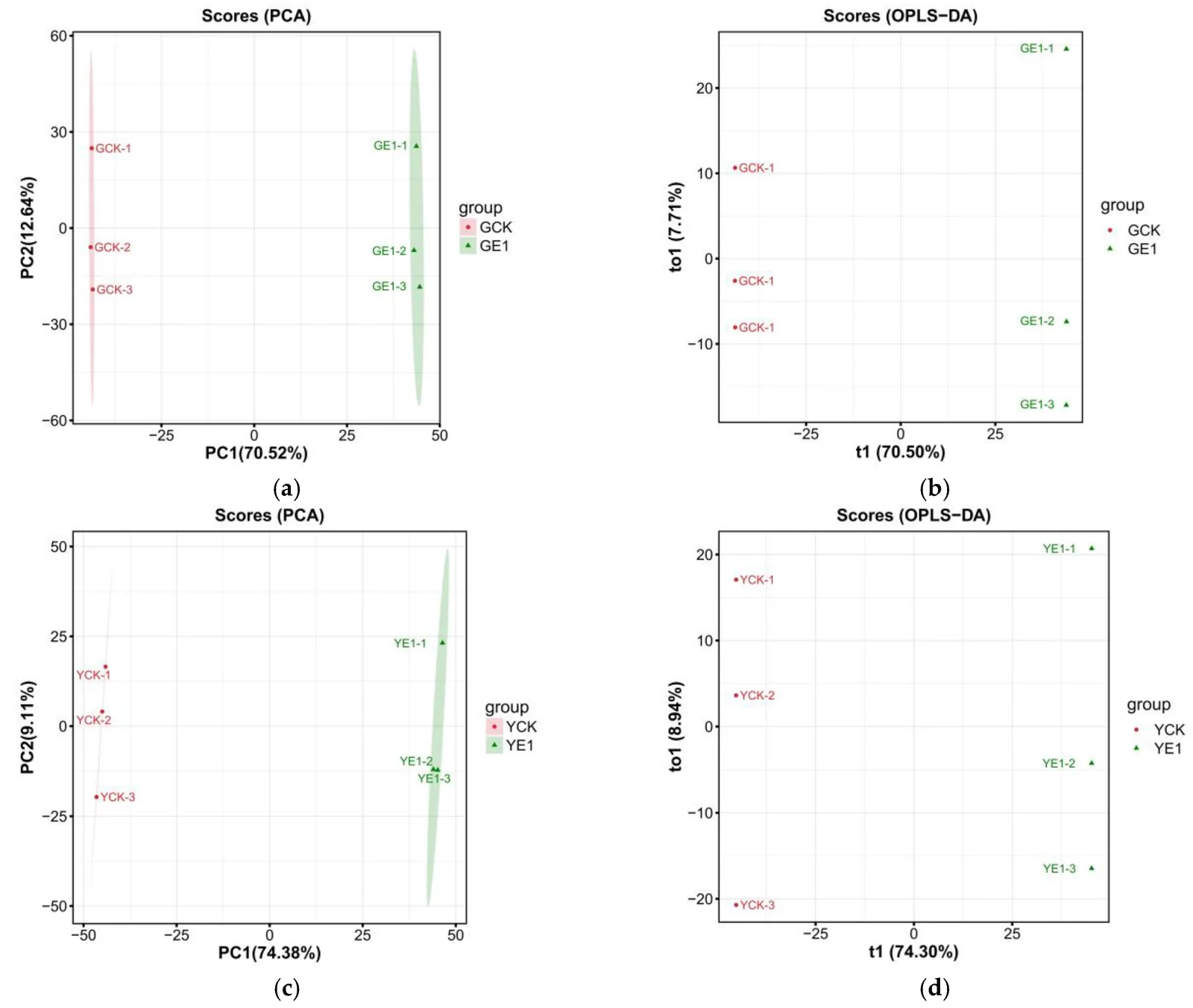
PCA and OPLS-DA plots for plant roots and leaves. (**a**) PCA plot for plant roots. (**b**) OPLS-DA plot for plant roots. (**c**) PCA plot for plant leaves. (**d**) OPLS-DA plot for plant leaves. CK, control; E1, fermentation-broth treatment; *n* = 3 independent biological replicates per treatment (each an individual plant); OPLS-DA models were validated by 200 permutation tests.

Contribution analyses using PCA and OPLS-DA on samples of plant roots and leaves revealed that root samples contributed 70.52% to PC1 and 12.64% to PC2 (Figure 5a); the two groups of samples showed a trend towards separation; visual separation in PCA was not treated as evidence of statistical significance. In the leaf samples, the contribution to PC1 was 74.38% and to PC2 was 9.11% (Figure 5c). To elucidate the patterns of metabolite changes in roots and leaves in response to the E1 fermentation preparation, this study employed the OPLS-DA method to integrate the types and abundances of compounds across different samples, with the aim of identifying differences in compound composition between different treatment groups. Based on the OPLS-DA analysis results (Figure 5b,d), it can be observed that root and leaf samples treated with the E1 fungal fermentation broth are distributed on the right-hand side of the confidence interval, whilst control group samples are distributed on the left-hand side; there is a strong tendency for these two sets of samples to be separated. Through PCA and OPLS-DA analyses, a clear separation between the control and experimental group samples in this study can be observed.

#### 3.4.2. Differential Metabolite Analysis

**Figure 6:**
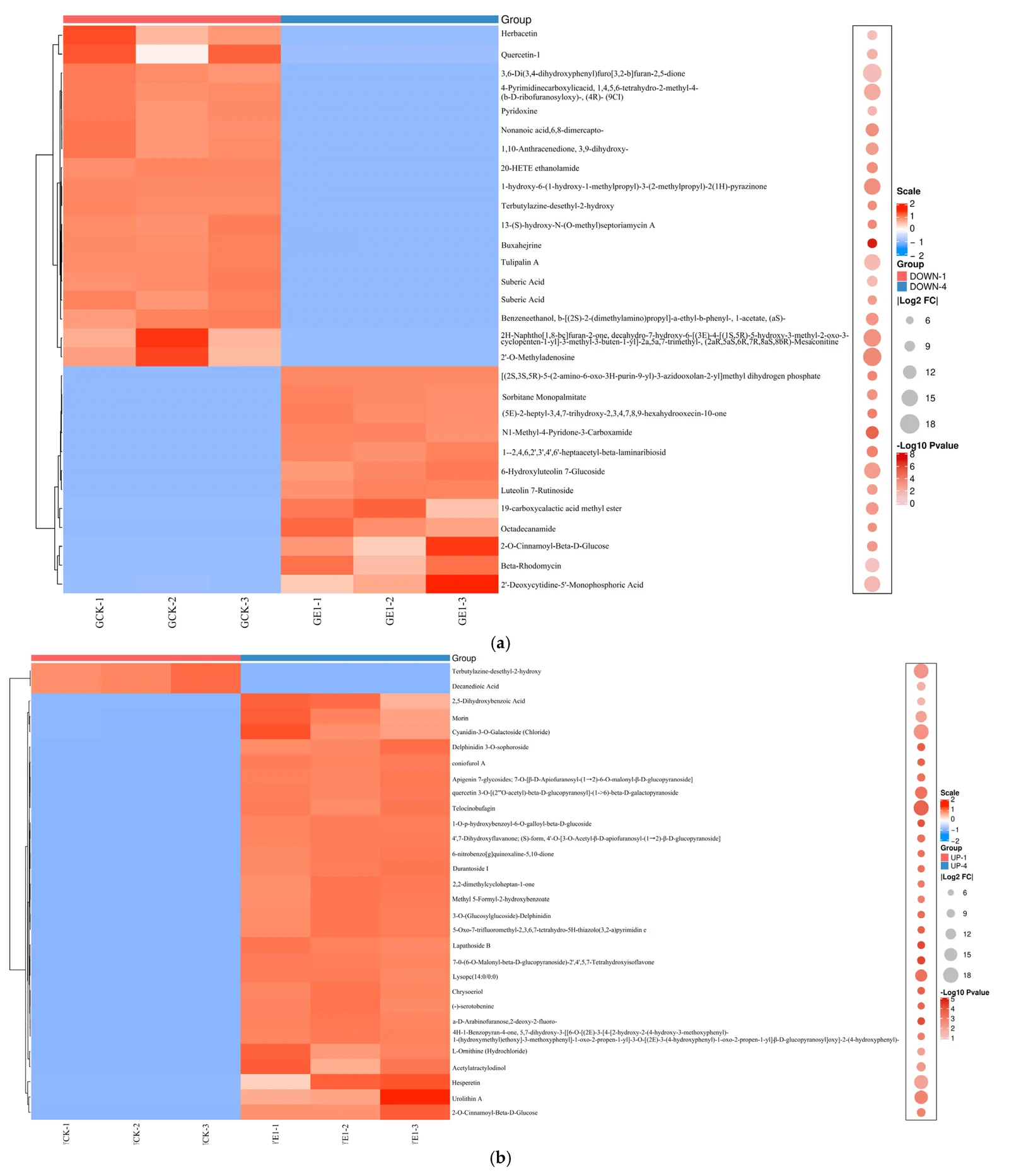
Heatmaps of DAMs. (**a**) Metabolite heatmap of the root system. (**b**) Metabolite heatmap of the leaves. CK, control; E1, fermentation-broth treatment; G, root tissue; Y, leaf tissue; *n* = 3 independent biological replicates per treatment (each an individual plant). DAMs as |log_2_FC| ≥ 1, *p* < 0.05 and VIP > 1.

This study constructed a multidimensional bubble heatmap of differentially accumulated metabolites (DAMs) that integrates the fold change and statistical significance. Based on pre-set dual screening thresholds for both significance and fold change, a large number of significantly DAMs were identified from the full-spectrum root metabolite library, covering characteristic secondary metabolites such as flavonoids, functional metabolites such as alkaloids, hydroxylated lipid derivatives, organic acids such as dicarboxylic acids, nucleoside phosphate derivatives, polyketone natural products, and other key components at critical nodes in multiple core primary and secondary plant metabolic pathways (Figure 6a). Among these, the metabolite exhibiting the highest upregulation fold was the acylated glycoside secondary metabolite 2-O-Cinnamoyl-β-D-Glucose, derived from the phenylpropanoid metabolic pathway. The second most significantly increased metabolite was octadecanamide from the octadecanoid pathway. N1-Methyl-4-pyridone-3-carboxamide also exhibited showing a strong upward trend. Substances such as fructoheptasaccharide, luteolin 7-rutinoside and 6-hydroxyluteolin 7-glucoside also exhibited a strong upward trend. The metabolite most significantly downregulated and associated with plant growth was tulipalin A. Suberic acid also showed a significant downward trend.

In the leaves, the metabolites identified based on the differentiation threshold encompassed multiple plant secondary metabolic pathways, including anthocyanins, flavonoid glycosides, phenolic acid derivatives, alkaloids and lipid derivatives. Lysopc (14:0/0:0) exhibited a marked upregulation trend (Figure 6b). Next is L-Ornithine. Propanoyl phosphate also exhibited a significant upward trend.

#### 3.4.3. KEGG Enrichment Analysis of Metabolomics

**Figure 7:**
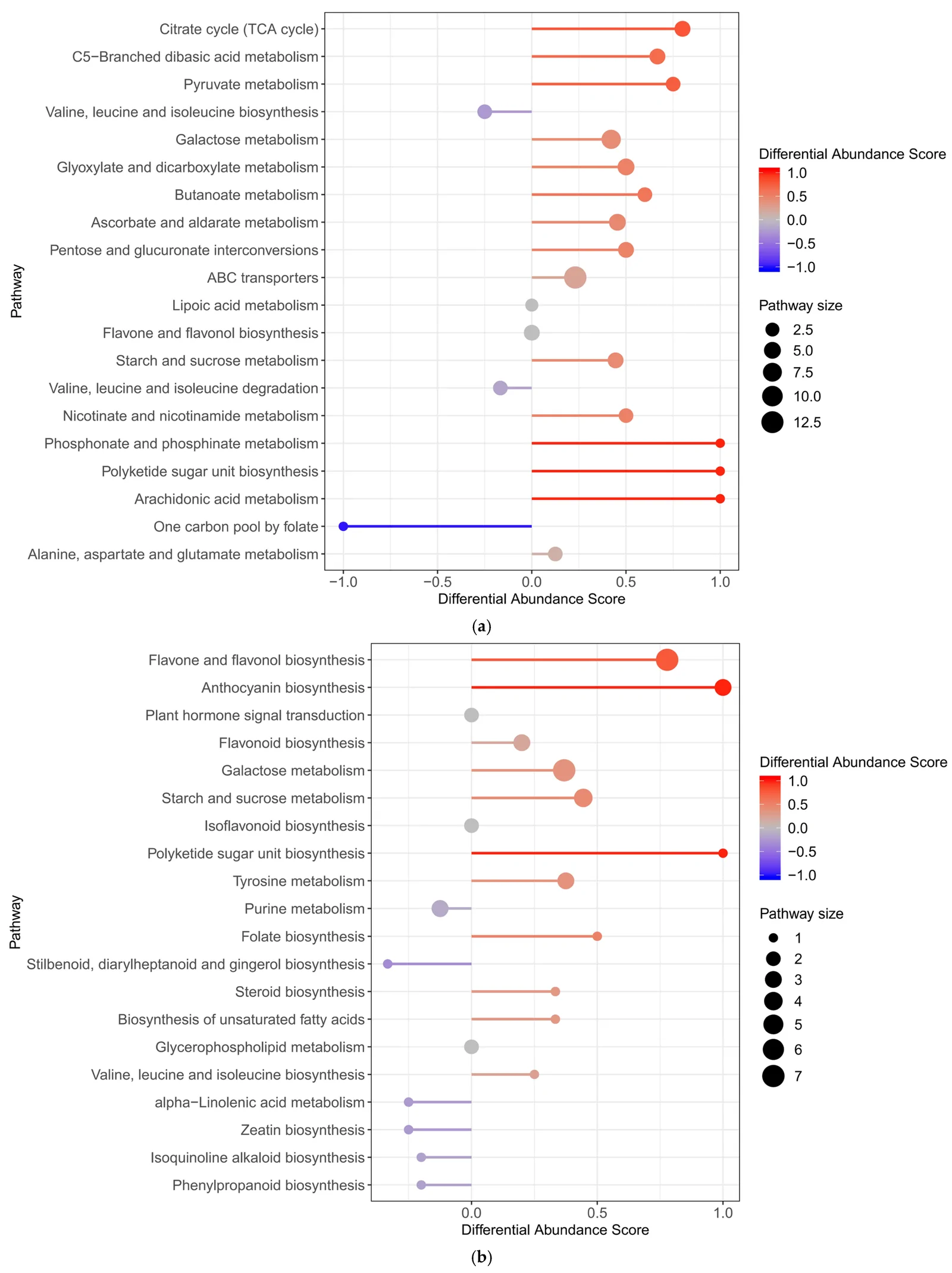
Differential metabolic pathway diagrams. (**a**) Differential metabolic pathway diagram for roots. (**b**) Differential metabolic pathway diagram for leaves. CK, control; E1, fermentation-broth treatment; G, root tissue; Y, leaf tissue; *n* = 3 independent biological replicates per treatment (each an individual plant). DAMs as |log_2_FC| ≥ 1, *p* < 0.05 and VIP > 1.

Through the screening and analysis of DAMs across different treatment groups, combined with KEGG functional annotation and pathway enrichment analysis, this study examined the metabolic pathways in fungal fermentation broths that may potentially promote plant growth. In the roots, the majority of these DAMs belong to key metabolic pathways such as the ABC transporter family, flavonoid biosynthesis (including the biosynthesis of flavonoids and their derivatives), and the TCA cycle (Figure 7a). Meanwhile, ATP-binding cassette (ABC) transporters are crucial transmembrane transporters capable of maintaining intracellular homeostasis. Furthermore, the upregulation of fundamental metabolic pathways—including the galactose metabolic pathway, the ascorbic acid/dehydroascorbic acid cycle, and the tricarboxylic acid cycle. In leaf samples treated with the fungal fermentation broth, the biosynthetic pathways for flavonoids and their derivatives exhibited the highest degree of enrichment, ranking first among all pathways analysed (Figure 7b). Concurrently, galactose metabolism and starch/sucrose metabolism also showed a high degree of enrichment.

### 3.5. Transcriptomic-Metabolomic Co-Enrichment

#### 3.5.1. KEGG Co-Enrichment Analysis of DEGs and Metabolites

Co-enrichment analysis of the transcriptome and metabolome revealed that the transcriptome and metabolome of the roots of *L. perenne* exposed to fungal fermentation broth were jointly enriched in only one pathway: ‘Starch and sucrose metabolism’. The starch and sucrose metabolism pathway was enriched with 7 DEGs and 4 DAMs. The transcriptomic and metabolomic data from the leaves were jointly enriched in ‘Phenylpropanoid biosynthesis’ and ‘Starch and sucrose metabolism’. These two metabolic pathways were jointly enriched with 6 DEGs and 8 DAMs. An analysis of the DEGs and metabolites enriched in the aforementioned pathways is shown in Table A6. In the roots, the DEGs *CIN*1, *SPS*3 and *BGLU*12 were up-regulated, whilst the expression levels of Alpha-glucosidase, *At*2g27500, *PDCB*4 and *At*3g13560 were down-regulated. In the two pathways in the leaves, only the *PAR* gene was down-regulated.

#### 3.5.2. Correlation Analysis Between DEGs and DAMs in Core Co-Enriched Pathways

**Figure 8:**
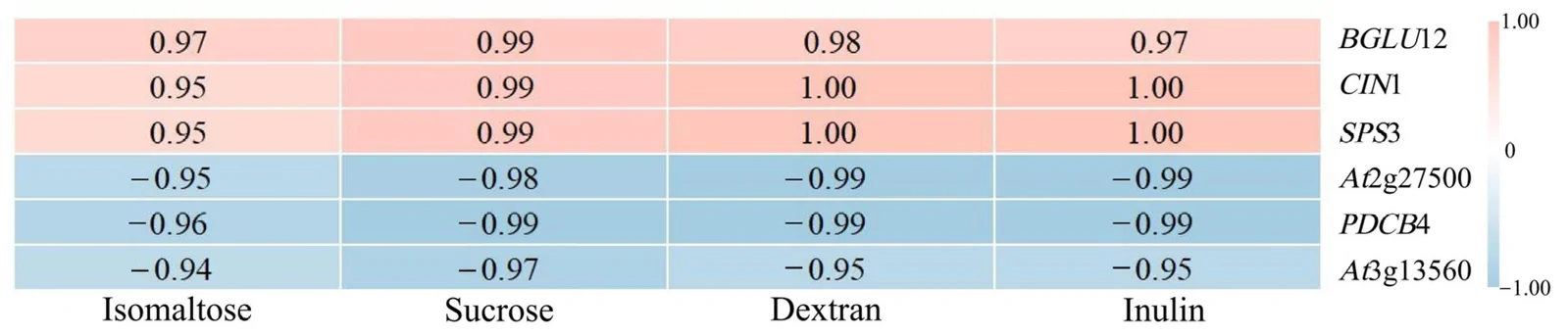
Correlation analysis between DEGs and DAMs in co-enrichment pathways within plant root systems. *n* = 6 (3 CK + 3 E1); defined Spearman correlation; stated BH-FDR correction and threshold (|r| ≥ 0.90, adj.*p* < 0.05).

**Figure 9:**
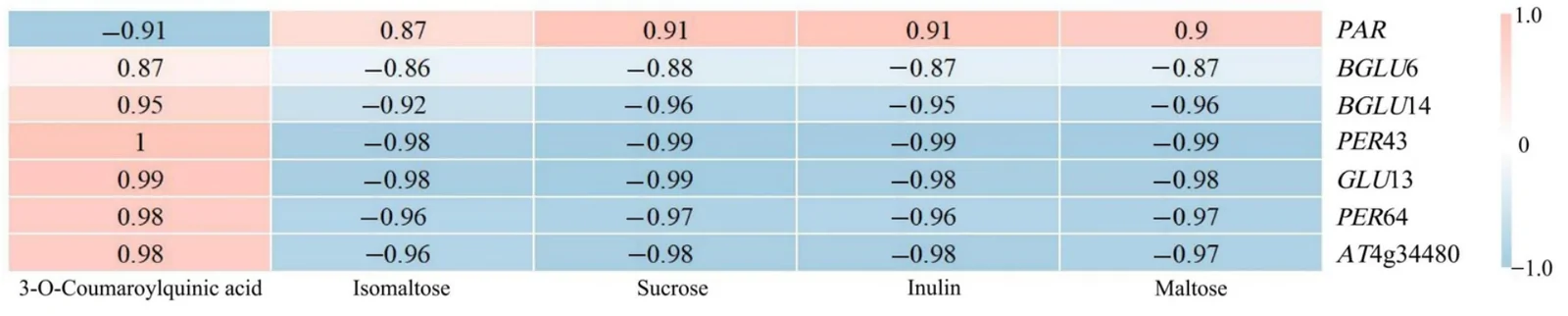
Correlation analysis between DEGs and DAMs in co-enriched pathways in plant leaves. *n* = 6 (3 CK + 3 E1); defined Spearman correlation; stated BH-FDR correction and threshold (|r| ≥ 0.90, adj.*p* < 0.05).

The correlation analysis of DEGs and metabolites in the co-enriched pathways of the root tissue is shown in Figure 8. Correlations between DEGs and metabolites were calculated using Spearman’s method, and *p* values were adjusted for multiple testing using the Benjamini–Hochberg procedure (FDR < 0.05). Some DEGs exhibited significant positive or negative correlations with multiple metabolites. The DEGs *SPS*3, *CIN*1 and *BGLU*12 showed strong positive correlations with the metabolites isomaltose, dextran, inulin and sucrose. These relationships are descriptive associations only: the direction of causality cannot be inferred from correlation data, and the functions of these genes were not experimentally validated in this study. We therefore present, as a tentative working hypothesis rather than a demonstrated mechanism, the possibility that upregulation of SPS3, CIN1 and BGLU12 may be associated with the changes in starch and sucrose metabolism and with the biomass accumulation observed in the pot experiment. Testing this hypothesis would require functional experiments (e.g., gene silencing or overexpression) that were beyond the scope of the present study. As shown in Figure 9, a correlation analysis of co-enriched DEGs and DAMs in leaves revealed that only the metabolite 3-O-coumaroylquinic acid showed a significant positive correlation with *BGLU*14, *PER*43, *BGLU*6, *GLU*13 and *At*4g34480.

## 4. Discussion

Most previous research on Fusarium has focused on its pathogenic effects on crops. Through ITS rDNA molecular analysis, a Fusarium strain was isolated from healthy *C. uncinatum* plants; its ITS rDNA sequence showed 99% similarity to that of *Fusarium napiforme*. In this study, we found that the fungal fermentation broth promotes the growth of *L. perenne*. By combining multi-tissue transcriptomics with non-targeted, broad-spectrum metabolomics analysis, this study provides candidate molecular pathways through which the fermentation broth of this *Fusarium* sp. isolate may promote host plant growth. This provides a partial theoretical basis and germplasm resource support for the development of functional microbial inoculants specifically targeted at forage crops.

### 4.1. Potential of the Fermentation Preparation of the Fusarium sp. Isolate as a Microbial Inoculant

The phenotypic quantification results of this study confirm that the growth-promoting effect of the fermentation preparation of this fungal isolate on *L. perenne* holds significant potential for agronomic applications: following treatment of the seeds with the fermentation broth of this fungus, the germination rate of *L. perenne* seedsincreased from 61.5% in the control group to 82%; during subsequent pot-grown cultivation cycles, compared with the control group, the host plants increased by 20.0%, 30.6% and 67.6% in height, root length and dry weight (*p* < 0.05), whilst fresh weight showed a non-significant trend (+22.5%, *p* = 0.136); It is worth noting that the increase in dry matter accumulation far exceeded the average effect values reported for most endophytic fungi of forage grasses in previous studies [46,47]. Conventional Fusarium strains are generally regarded as pathogenic fungi that adversely affect plant growth and development [48]. In this study, unlike the nutrient deficiency effects typically caused by conventional *Fusarium* strains when infecting hosts, the fermentation broth of this fungal strain did not cause any visible pathological damage to the host; on the contrary, the fermentation broth significantly enhanced the growth and development of *L. perenne*. These results suggest that this fermentation preparation warrants further evaluation as a candidate microbial inoculant.

### 4.2. Fermentation Broth from a Fungal Isolate of the Genus Fusarium May Accelerate the Growth and Development

The tissue-specific transcriptomic results for roots and leaves in this study indicate that the host’s molecular response to fungal fermentation broth exhibits significant tissue- and spatial-specificity: As the root system serves as the core functional region for the uptake of nutrients, the total number of annotated DEGs was 314 higher than in the leaves; at the biological process level, these DEGs were significantly enriched in carbohydrate metabolism, oxidative stress response, hydrogen peroxide degradation, cell wall organisation, and the cinnamic acid biosynthesis pathway. Of these, 158 DEGs were enriched in carbohydrate metabolism, whilst 797 up-regulated genes were enriched in membrane-integrated components. The substantial up-regulation of peroxidase family genes not only scavenges low levels of reactive oxygen species (ROS) generated during exposure to the fermentation broth but also may contribute to root cell wall extension and lignification and is consistent with the sustained elongation of the host root system [49,50]. The transcriptional response in leaf tissues, meanwhile, shifted towards the maintenance of photosynthetic function and the enhancement of defence capabilities; DEGs were significantly enriched in cutin-cork-wax biosynthesis, starch and sucrose metabolism, and plant–pathogen interaction pathways, with 146 DEGs enriched in chloroplast functional components. It is worth noting that, during this process, the plant’s immune response pathways may be moderately activated, but do not trigger a typical hypersensitive necrotic immune response, which may be associated with a shift of carbon resources towards vegetative growth and biomass accumulation; this remains a hypothesis.

Quality control results based on PCA and OPLS-DA confirmed that root and leaf samples from the control and treatment groups were clearly separated, indicating that this fungal fermentation broth may alter the endogenous metabolome of *L. perenne*. The most highly up-regulated differentially expressed metabolite in the root system was 2-O-cinnamoyl-β-D-glucose, derived from the phenylpropanoid pathway [51]. As an acyl donor precursor, this compound may be involved in the acylation of cell wall polysaccharides and lignin monomers, which is consistent with the up-regulation of genes in the cell wall organisation pathways identified in the transcriptome [52]. The differential metabolite profile in the leaves is consistent with the photosynthetic requirements of the aboveground parts: The content of LysoPC (14:0/0:0), which is involved in the intracellular organelle-to-organelle transport of lipids, was significantly up-regulated, which may facilitate the directed transport of long-chain fatty acids between the endoplasmic reticulum and chloroplasts and may contribute to the supply of precursors for the biosynthesis of photosynthetic membrane lipids [53]; the significant up-regulation of ornithine, may be associated with the coordinated regulation of vegetative growth in both the underground root system and the aboveground parts [54]. This suggests that the fungal fermentation broth may influence substance transport within plants by altering the levels of endogenous plant hormones.

### 4.3. Possible Mechanisms by Which the Fermentation Broth Promotes Plant Growth

A co-enrichment analysis combining transcriptomics and metabolomics identified two potential core functional regulatory pathways involved in this growth-promoting process: in the root system, only the starch and sucrose metabolic pathways were co-enriched; whereas in the leaves, the phenylpropanoid biosynthesis pathway was co-enriched alongside the starch and sucrose metabolic pathways, suggesting candidate regulatory pathways associated with growth promotion. Three genes—SPS3, CIN1 and BGLU12—showed strong positive correlations (|r| > 0.9, BH-adjusted *p* < 0.05) with the downregulated storage carbohydrates (such as isomaltose, glucan, inulin and sucrose) in this metabolic pathway. We hypothesise that this may facilitate the conversion of polysaccharide carbon sources into monosaccharide substrates available for cell growth; however, this remains to be tested experimentally [55,56,57]. In the phenylpropanoid biosynthesis pathway, which was simultaneously enriched in the leaves, several up-regulated genes from the peroxidase family were co-expressed with the up-regulated 3-O-coumaroylquinic acid. This may provide ample phenolic acid precursors for lignin synthesis in the leaf cell walls, which may contribute to the stability of the photosynthetic structures [58].

### 4.4. Limitations of the Study

The present study has several limitations that should be considered when interpreting these results. First, the isolate was identified on the basis of ITS rDNA only, which does not reliably resolve species within the *Fusarium fujikuroi* species complex; multi-locus sequencing (TEF1-α and RPB2) is required before a species name can be assigned, and the strain is therefore referred to as a *Fusarium* sp. isolate. Second, the treatment consisted of root irrigation with a fungal fermentation broth rather than inoculation with the fungus itself. Colonisation of *L. perenne* tissues was therefore not verified, and the observed responses cannot be attributed to a symbiotic or mutualistic interaction. Third, the effects of the broth could not be fully separated from those of residual medium components, changes in rhizosphere pH, or fungal metabolites. Fourth, no pathogenicity re-inoculation test, mycotoxin quantification (e.g., fumonisins), or field trial was performed; the absence of visible disease symptoms under our experimental conditions does not establish that the strain is non-pathogenic or safe. Notably, the pot experiment included a water control only and no medium-only control; the effects observed in the pot experiment are therefore attributed to the fermentation preparation as a whole and cannot be assigned specifically to the living fungus or to any single component of the broth. Finally, carbon flux, photosynthetic parameters, enzyme activities and hormone concentrations were not measured, and the proposed mechanisms therefore remain hypotheses requiring functional validation. The correlations between gene expression and metabolite accumulation reported here are descriptive and indicate a statistical association only; they do not by themselves establish a causal relationship, since no functional validation was performed in this study. In particular, our data do not show that upregulation of SPS3, CIN1 and BGLU12 causes the observed increase in biomass, and these genes are therefore discussed as candidate markers of the growth response rather than as its cause. We present this interpretation as a working hypothesis to be tested by functional experiments, and not as a demonstrated mechanism of action.

## 5. Conclusions

A *Fusarium* isolate was obtained from healthy *C. uncinatum* plants; it caused no visible disease symptoms under the conditions tested, and its fermentation preparation promoted the growth of *L. perenne*. The fermentation broth of this fungal strain increased the seed germination rate of *L. perenne* from 61.5% to 82% and significantly promoted biomass accumulation—with dry matter content increasing by up to 67.6%—without causing any pathological damage throughout the process. Integrated transcriptomic and metabolomic analyses identified candidate pathways, including starch and sucrose metabolism, that are associated with biomass accumulation in treated plants. Because carbon flux, enzyme activities and hormone levels were not measured, these pathways are proposed as hypotheses rather than demonstrated mechanisms of action, and functional validation is required. These findings identify a candidate fungal isolate for the future development of microbial inoculants for forage crops; however, any such application would be conditional on a definitive multi-locus taxonomic identification of the strain (TEF1-α and RPB2) and on a comprehensive biosafety assessment, including pathogenicity re-inoculation tests, mycotoxin quantification and field trials.

## Data Availability

The original data presented in this study are not publicly available, owing to institutional data-sharing restrictions and because the dataset forms part of an ongoing research programme. The datasets are available from the first author.

## Appendix A

**Table A1:** Transcriptome sequencing and analysis parameters.

Parameter	Detail/Value
RNA integrity (RIN)	8.6–9.4 (all samples ≥ 8.5; Agilent 2100 Bioanalyzer)
Library preparation	Illumina TruSeq Stranded mRNA Library Prep Kit; poly(A) enrichment
Sequencing platform & mode	Illumina NovaSeq 6000, paired-end 150 bp (PE150)
Total clean data	5.79–6.95 Gb per sample
Sequencing quality (Q30)	95.63–96.78% per sample
Read processing	Trimmomatic: adapter removal, quality trimming (Phred Q ≥ 20), removal of reads with >10% N bases
Reference genome	Lolium multiflorum (Italian ryegrass) reference genome assembly
Alignment software	HISAT2 v2.2.1
Mapping rate	61.58–68.64% per sample (uniquely mapped reads: 57.0–62.2%)
Transcript assembly & quantification	StringTie v2.2.1; gene-level FPKM (Fragments Per Kilobase per Million mapped reads)
Normalization	DESeq2 median-of-ratios normalization (for DE analysis); FPKM for expression level comparison
Differential expression	DESeq2 v1.38.0; negative binomial generalized linear model; Wald test
DEG threshold	|log_2_Fold Change| ≥ 1 (i.e., FC ≥ 2 or ≤ 0.5) and FDR < 0.01
Novel gene discovery	19,721 novel loci identified via StringTie; 5419 functionally annotated
Raw data availability	Not deposited in a public repository; raw reads, processed expression matrices, metadata and analysis code are available from the first author on reasonable request.

**Table A2:** GO/KEGG enrichment analysis parameters.

Parameter	Detail/Value
Background gene set	All annotated genes in the L. multiflorum reference genome (including 19,721 novel genes with functional annotation)
Enrichment test	Hypergeometric test (over-representation analysis)
Software	ClusterProfiler v4.6.2 (R package)
GO annotation source	Combined: NCBI nr, Swiss-Prot, Pfam, and eggNOG annotations mapped to GO terms via Blast2GO pipeline
KEGG annotation source	KEGG Automatic Annotation Server (KAAS) + blastx against KEGG GENES database
Multiple-testing correction	Benjamini–Hochberg (BH) FDR procedure; q-value reported
Significance threshold	Adjusted *p*-value (q-value) < 0.05 for both GO terms and KEGG pathways
Enrichment visualization	Dot plots (GeneRatio vs. −log_10_(q-value)), bar plots, and enrichment chord plots; top 20 most significant terms/pathways shown
Additional enrichment analysis	Gene Set Enrichment Analysis (GSEA) using all genes ranked by log_2_FC; GO and KEGG gene sets; permutation-based FDR

**Table A3:** Metabolomic analysis parameters.

Parameter	Detail/Value
Biological replicates	*n* = 3 independent biological replicates per treatment (each an individual plant); matched with transcriptomics
Metabolomics platform	UPLC (Waters I-Class PLUS) + AB Sciex QTRAP 6500+; ESI±; MRM mode
Metabolite database	GB-PLANT database; 3,484 metabolites detected
Quality control (QC)	Pooled QC every 10 injections; >85% metabolites QC CV < 0.5
Missing values	>50% missing → removed; remaining → LOD/2 imputation
Normalization	Internal-standard + TIC normalisation
Transformation	UV scaling (multivariate); log_2_ transform (univariate)
Metabolite identification	Level 1 (>90%)/Level 2 (>80%)/Level 3 (>60%)
Univariate statistical analysis	Student’s two-tailed *t*-test on log_2_ data; *p* < 0.05
Multivariate statistical analysis	PCA + OPLS-DA (ropls v1.30.0); 7-fold CV
OPLS-DA model performance	R^2^Y = 0.99, Q^2^ = 0.95
OPLS-DA permutation test	200 permutations; Q^2^ intercept = −0.32, *p* < 0.005
Variable Importance in Projection (VIP)	VIP from first predictive component; VIP > 1
Differential metabolite criterion	|log_2_FC| ≥ 1 AND *p* < 0.05 AND VIP > 1
Multiple-testing correction	BH FDR; FDR-adjusted *p* < 0.05 for KEGG enrichment
KEGG pathway enrichment	Hypergeometric test + BH FDR; adjusted *p* < 0.05

**Table A4:** Seed germination rate.

Group	Dish 1	Dish 2	Dish 3	Dish 4	Overall Rate	OR_MH (95% CI)/*p*
CK-G	31/50	32/50	29/50	31/50	123/200	—
E1	43/50	41/50	39/50	41/50	164/200	2.86 [1.80, 4.53] *p* = 5.7 × 10^−6^

**Table A5:** Physiological data.

Trait	CK (Mean ± SD)	E1 (Mean ± SD)	Change (%)	t (df)	*p*-Value	Cohen’s d [95% CI]
Average plant height/cm (*n* = 10/10)	28.17 ± 4.2	33.80 ± 4.72	+20.0%	2.82 (17.8)	0.011	1.26 [0.30, 2.22]
Average root length/cm (*n* = 10/10)	7.72 ± 1.96	10.08 ± 2.28	+30.6%	2.48 (17.6)	0.024	1.11 [0.17, 2.05]
Average Fresh weight/g (*n* = 5/5)	0.74 ± 0.15	0.90 ± 0.16	+22.5%	1.66 (8.0)	0.136	1.05 [−0.27, 2.37]
Average dry weight/g (*n* = 5/5)	0.05 ± 0.02	0.09 ± 0.01	+67.6%	3.51 (7.1)	0.010	2.22 [0.65, 3.80]

**Table A6:** DEGs and metabolites in the co-enriched pathways of the *Fusarium* sp. isolate.

Location	Channel Name	DEGs	Increase/Decrease	DAMs	Increase/Decrease
Root	Starch and sucrose metabolism	CIN1	up	Dextran	down
		At2g27500	down	Inulin	down
		PDCB4	down	Sucrose	down
		SPS3	up		
		At3g13560	down		
		BGLU12	up		
Leaf	Phenylpropanoid biosynthesis	PER64	up	3-O-Coumaroylquinic acid	up
		PAR	down		
		BGLU14	up		
		PER43	up		
		BGLU6	up		
	Starch and sucrose metabolism	GLU13	up	Inulin	down
		At4g34480	up	Sucrose	down
		BGLU14	up	Maltose	down
		BGLU6	up	Isomaltose	down
